# Relationship of frozen-thawed semen quality with the fertility rate after being distributed in the Brahman Cross Breeding Program

**DOI:** 10.14202/vetworld.2020.2649-2657

**Published:** 2020-12-14

**Authors:** Berlin Pandapotan Pardede, Muhammad Agil, Yudi Yudi, Iman Supriatna

**Affiliations:** 1 Reproductive Biology Study Program, Faculty of Veterinary Medicine, IPB University, Bogor, Indonesia; 2 Department of Veterinary Clinic, Reproduction, and Pathology, Division of Reproduction and Obstetrics, Faculty of Veterinary Medicine, IPB University, Bogor, Indonesia

**Keywords:** artificial insemination, Brahman cross, distribution, fertility rate, frozen semen quality

## Abstract

**Background and Aim:**

Various factors can reduce the quality of semen used for artificial insemination and have an impact on fertility decline, such as poor handling during frozen semen distribution. This study was aimed at assessing the quality of frozen-thawed semen after distribution in the field and its importance in maintaining fertility.

**Materials and Methods:**

The Brahman Cross (BX) breeding program of PT Lembu Jantan Perkasa, Indonesia, was used. This program was preferred due to its adherence to guidelines that limit the effects of extraneous factors that may affect semen quality. Frozen-thawed semen samples from eight bulls with the same production code were analyzed and compared between the production site (artificial insemination [AI] center) and the field (BX breeding program). Total and progressive motility (PM) of sperm were determined using computer-assisted semen analysis. Plasma membrane integrity (PMI) was assessed using hypoosmotic swelling test, sperm viability using Eosin-Nigrosin staining, acrosome integrity using trypan blue-Giemsa staining, morphological abnormalities using William staining, and DNA fragmentation using toluidine blue staining. The fertility rate was determined using the conception rate (%) derived from AI data based on 502 AI services and 478 cows in the BX breeding program. A t-test was used to compare the quality of frozen-thawed semen before and after distribution. The relationship between the qualities of frozen semen after distribution in the field with fertility was analyzed using Pearson correlation.

**Results:**

There was no significant difference (p>0.05) in the quality of frozen-thawed semen (sperm motility, PMI, viability, acrosome integrity, abnormalities, and DNA fragmentation) between the production site (AI center) and after distribution in the field (BX breeding program). The semen met the minimum standards for AI programs. Total motility (r=0.986), PM (r=0.961), sperm viability (r=0.971), PMI (r=0.986), and acrosome integrity (r=0.992) were all positively correlated (p<0.05) with fertility rate; while sperm abnormalities (r=−0.996) and sperm DNA fragmentation (r=0.975) were negatively correlated (p<0.05) with fertility rate.

**Conclusion:**

The study showed that to achieve the maximal and optimal fertility rate in bulls in an AI program, the overall quality of frozen-thawed semen in all aspects is critical. This can be achieved if the handling during distribution and storage, as well as the various factors that may affect the quality of semen in the field, can be controlled properly.

## Introduction

Artificial insemination (AI) is one of the oldest reproductive technologies that have been proven to be capable of improving the genetic quality of livestock. This technology can produce a high quantity of high-quality offspring within a short period by utilizing as many superior bulls as possible [1]. The measure of the success of AI on a farm is a high frequency of pregnant cows indicating an improved reproductive efficiency [2]. Conception rate (CR) is one indicator of fertility that can be used to assess reproductive efficiency [3]. CR is the percentage of pregnant cows after the first insemination. Several factors can influence the success of AI and CR, namely, the quality of frozen semen, the inseminator, reproductive health of the cows [4], and the accuracy of breeders in detecting estrus. Dogan *et al*. [5] stated that the CR is correlated with the quality of semen. Mekonnen *et al*. [6] and Kebede [2] reported that good semen quality will greatly influence the success of AI and will increase the CR in the field [7]. Semen quality is an important factor in the success of fertilization of AI programs [8,9]. Good quality frozen semen must contain motile, living sperm with intact plasma membranes, intact acrosomes have good morphology, and DNA to fertilize oocytes [10-12].

The frozen semen that is used for AI in the field usually goes through various quality testing procedures at the production site (AI center) following applicable standards at each AI center. CRs in the field in Indonesia have not yet reached the desired levels. As observed by Rusdiana and Soeharsono [13] who report a relatively low CR in Indonesia of 57.68%, while Butler [14] states that desirable CR must be up to 70% of all first-inseminated cows. This has led to speculation regarding the factors that may be responsible for the observed low CR. Chief among these factors is the quality of frozen-thawed semen in the field. Several factors can cause a decrease in the quality of semen such as genetics [15], breed, bull weight, bull age [16], cryopreservation processes, and various other external factors, such as handling of frozen semen samples. The distribution of frozen semen samples is an important management aspect and can affect the quality of frozen semen. McDonald *et al*. [17] reported that there was a decrease in the quality of reproductive cell samples (frozen oocytes) in humans as a result of abnormal conditions and poor handling of samples during the distribution or transportation process. In addition, Parmegiani *et al*. [18] observed that the type of sample transportation used and the conditions when shipping or distributing frozen human oocyte samples will affect the quality of the oocytes. Similarly, Carrell *et al*. [19] reported that temperature fluctuations during the distribution and handling of frozen semen samples in humans will harm semen quality. This contrasts with Til *et al*. [20], who reported that the distribution or transportation of frozen semen in humans did not affect the quality of sperm. Ramírez-Reveco *et al*. [21] also reported that as long as the storage temperature of frozen samples in containers was maintained at −196°C, the quality of the frozen semen could still be maintained for a long time.

However, information regarding the quality of frozen semen after distribution is limited to human samples. Research related to the quality of frozen semen in livestock after distribution in the field, especially in bulls, requires further investigation. It is of importance to establish whether the distribution process will negatively affect the quality of frozen semen and consequently, field fertility. For this reason, this study was aimed at assessing the quality of frozen-thawed semen after distribution in the field and its importance in maintaining fertility.

## Materials and Methods

### Ethical approval

This study was conducted following the operational standards applicable in Indonesia, namely, SNI ISO 9001: 2015 No. 824 100 16072 at the Lembang AI Center and SNI ISO 9001: 2015 No. G.01-ID0139-VIII-2019 at the Singosari AI Center and supervised by a veterinarian from both AI Centers. Every stage of this study considered the principles of animal welfare, which refer to the ethical clearance requirements of the Animal Care and Uses Committee. This study used frozen semen samples obtained from a series of processes, including the process of semen collection from healthy bulls using an artificial vagina, which does not affect normal physiological processes.

### Study period and location

The research was conducted from April to October 2019 at PT Lembu Jantan Perkasa, Banten, Lembang AI Center, West Java, Singosari AI Center, East Java and the Laboratory of Animal Reproduction, Breeding and Cell Culture, Research Center for Biotechnology, Indonesian Institute of Sciences, West Java, Indonesia.

### Experimental animals

Considering that many factors can influence the success of AI processes in the field outside of semen quality, such as inseminator skills, reproductive health of cows, and the accuracy of estrus detection, the study focused on one frozen semen distribution location, namely, the Brahman Cross (BX) breeding program of PT Lembu Jantan Perkasa (PT LJP). PT LJP is a fattening company in Indonesia, founded in 1990, which has been involved in the BX breeding program since 2004. The BX breeding program is supervised by veterinarians and trained officers so that the reproductive health of cows to be used for insemination is well controlled, estrus can be predicted and observed accurately, and the insemination process can also be done timely and properly. Cows with reproductive disorders are not used in the breeding program and are transferred to the fattening program. The BX cows will be inseminated using frozen semen distributed from the National AI Center in Indonesia, which uses four bull breeds: The Brahman, Simental, Limousine, and Bali. The semen is distributed in liquid nitrogen containers with a capacity of 45 L. Before and after distribution, sperm motility is checked in multiple samples using a light microscope and following the Indonesian National Standard for sperm motility (>40% motility).

The first stage of the study was checking the AI records of each bull used in the BX breeding program, which was then followed by checking the availability of frozen semen in the storage containers at the BX breeding program. This was followed by tracking frozen semen with the same production code at the production site (AI center) that will then be used for further analysis. Production code is a code on a straw which contains information related to the year and serial number of frozen semen production from a bull. Six frozen semen straws from eight bulls (B1-B8) with the same production code were available, both at the BX breeding program site and at the production site (AI center). These were used for the assessment of semen quality before and after distribution to the BX breeding program to determine if there were any changes in semen quality. The thawing of frozen semen was carried out in a water bath at 37°C for 30 s. The assessment done included the following parameters: Sperm motility, plasma membrane integrity (PMI), sperm viability, acrosome integrity, sperm abnormalities, and sperm DNA fragmentation.

### Sperm motility

Computer-aided sperm motility assessment was carried out using the SpermVision Program (Minitüb, Tiefenbach, Germany), and paired with Carl Zeiss Microimaging GmbH (Gottingen, Germany) equipped with a warm stage at 38°C. Aliquots (6 μL) of semen samples were dropped on glass slides and covered with a slipcover. The kinematics of around 750-1000 sperm cells in a total of five fields were evaluated using the SpermVision software program with specific settings for bull sperm. The kinematic parameters used are total motility (TM) and progressive motility (PM).

### PMI

PMI was evaluated using a hypoosmotic swelling test (HOS) [22]. A total of 20 μL of semen samples were inserted into a microtube containing 300 μL of HOS solution (0.735 g Na citrate, 1351 g Fructose, and 100 mL distilled water [DW]). The semen-HOS solution mixture was incubated in a water bath at 37°C for 30 min. Examination of the integrity of the cryopreserved semen membrane was carried out after incubation by placing a drop of the semen-HOS solution mixture on an object-glass, then covered it using a slipcover and observed with a light microscope with 40× objective. Sperm with intact PMI show a reaction in the form of a bulging or circular tail. The percentages of sperm that reacted and those that did not react were calculated from a total of at least 200 sperm cells.

### Sperm viability

Sperm viability was assessed using Eosin-Nigrosin staining. A total of 10 μL of semen was placed on a glass object, then 20 μL Eosin-Nigrosin staining solution was added and homogenized. Sperm smear preparations from the mixture were made and dried on a heating table. Two hundred cells per slide of non-stained live sperm (transparent head) and dead sperm (redhead) were evaluated by a light microscope with a 40× objective [23].

### Acrosome integrity

Assessment for acrosome integrity was carried out using trypan blue-Giemsa staining. The staining began by dripping semen samples and 0.2% trypan blue solution (0.4% trypan blue [Sigma T8154] and 0.9% NaCl [1:1]) simultaneously. The mixture was then homogenized. Smear preparations were made and dried vertically. The preparation was fixed vertically in a staining jar for 2 min in a neutral red solution (86 mL 1.0 N HCl, 14 mL formaldehyde 37%, 0.2 g neutral red [Merck, Darmstadt, Germany]). The preparations were rinsed in DW and then stained with 7.5% Giemsa (Merck, Darmstadt, Germany) in a vertical staining jar for 12-20 h (overnight) at room temperature. Preparations were then rinsed under running water, then dipped in DW for 2 min, and dried [24]. The assessment was carried out on 200 cells using a light microscope with a 40× objective. The head of sperm with an intact acrosome will stain purple, while sperm with a non-intact acrosome will be seen as pale or faded lavender.

### Sperm abnormalities

Morphological observations of sperm were carried out using a modified carbol-fuchsin-eosin (William) staining method [25]. The smear preparations of the semen samples were fixed using Bunsen, then dipped in absolute alcohol for 3-4 min, then removed and dried. The dried slides were rinsed using a 2% chloramine solution for approximately 2 min until all mucus was removed and the semen smear looked clean. The slides were then washed with DW, followed by 95% alcohol, and then stained with carbol-fuchsin-eosin staining solution for approximately 6 min. After this, the slides were washed under running water and dried. Five hundred cells per slide were assessed using a light microscope with a 40× objective.

### Sperm DNA fragmentation

The determination of DNA fragmentation can be done indirectly by evaluating the structural changes within the chromatin. The examination of chromatin structure was carried out using the toluidine blue (TB) staining technique [26]. The TB staining method began by making a smear sample of the semen on an object-glass. The preparation was dried and fixed by ethanol 96%:acetone (1:1) for 60 min at 40°C. After the fixation, the preparation was dried before being hydrolyzed in 0.1 N HCl for 5 min at 4°C. Then, 0.05% TB staining (dissolved in 50% MCllvaine’s citrate-phosphate buffer, pH 3.5) was carried out for 5 min at room temperature (20-25°C) after which the preparation was rinsed 3 times using DW. The preparation was stained, rinsed again with DW, and observed. Sperm heads with good chromatin integrity will be bright blue, while the sperm with fragmented chromatin will be dark blue. Five hundred cells per slide were assessed by a light microscope with a 40× objective.

### Fertility rate

The fertility rate for each bull was determined using the success data of AI in the BX Breeding Program. The CR is the percentage of cows becoming pregnant in one insemination procedure relative to the number of cows inseminated. The fertility rate was obtained based on 502 AI services and 478 receptor cows in the field for each bull (n=8). Pregnancy diagnosis was carried out transrectally at 45-60 days after AI.

### Statistical analysis

A t-test was used to compare the quality of frozen-thawed semen before and after distribution. The relationship between frozen-thawed semen quality and fertility rate was determined using Pearson correlation analysis and linear regression. All tests used a significance level of p<0.05. These statistical analyses were performed using SPSS ver. 25.0 (IBM, Armonk, NY, USA) and Sigma Plot for Windows ver. 14.0 (Dundas Software Ltd, Germany). The results obtained were tabulated and presented in a mean±standard deviation format.

## Results

There was no significant difference in total and progressive sperm motility (p>0.05) in frozen-thawed semen before and after distribution to the BX breeding program, with a range of 45-81% (Table-1). The sperm PMI in frozen-thawed semen did not differ significantly before and after distribution to the BX breeding program (p>0.05) and ranged between 62% and 84% (Table-1).

**Table-1:** The results of the analysis of TM, PM, and PMI in frozen semen before and after distribution.

Bulls (n=8)	TM (%)		PM (%)		PMI (%)	
		
	AI center	BX-bp	AI center	BX-bp	AI center	BX-bp
B1	66.47±0.80	65.89±0.74	52.85±0.35	52.23±0.52	71.08±0.01	70.08±0.01
B2	61.48±2.93	60.69±2.85	48.74±1.00	47.82±0.77	66.17±0.03	65.17±0.03
B3	60.64±1.71	59.92±1.70	46.79±0.57	45.93±0.55	63.58±0.01	62.79±0.01
B4	75.26±3.02	74.71±2.95	67.12±2.29	66.43±2.19	78.42±0.01	77.42±0.01
B5	82.01±1.03	81.30±0.91	72.16±2.26	71.12±2.53	85.58±0.01	84.58±0.01
B6	80.26±0.31	79.63±0.32	69.58±2.44	68.68±2.58	82.83±0.01	81.83±0.01
B7	71.42±2.23	70.64±2.19	54.82±1.66	54.06±1.51	74.00±0.02	73.00±0.02
B8	65.69±1.48	64.81±1.41	50.05±1.06	49.24±0.90	69.00±0.01	68.00±0.01

B1-B8=Bull-1…bull-8, AI=Artificial insemination, BX-bp=Brahman Cross breeding program, TM=Total motility, PM=Progressive motility, PMI=Plasma membrane integrity

**Figure-1:**
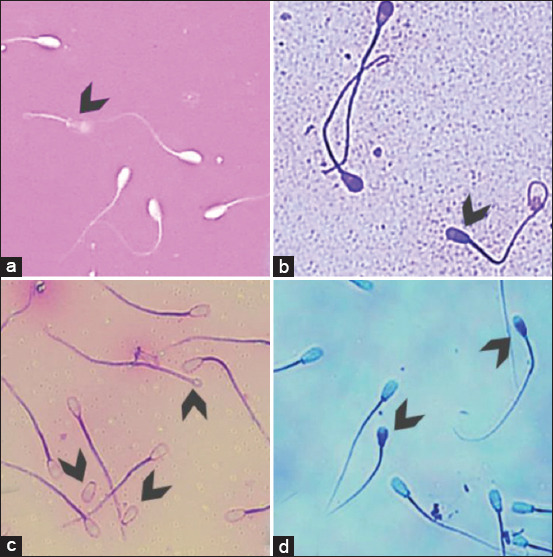
(a) Sperm viability assessment by Eosin-Nigrosin staining. The living sperm showed a colorless-head; the dead sperm will be colored by eosin and showed a redhead (arrow). (b) Sperm acrosome integrity assessment by trypan blue-Giemsa staining. Sperm with an intact acrosome showed a purple head, while a non-intact acrosome showed a pale or faded lavender head (arrow). (c) Sperm morphology assessment by carbol fuchsin eosin staining to distinguish between normal and abnormal sperm (arrow). (d) Sperm DNA fragmentation assessment by toluidine blue staining. Non-fragmented sperm showed a bright blue head, while fragmented sperm showed a dark blue head (arrow).

**Figure-2:**
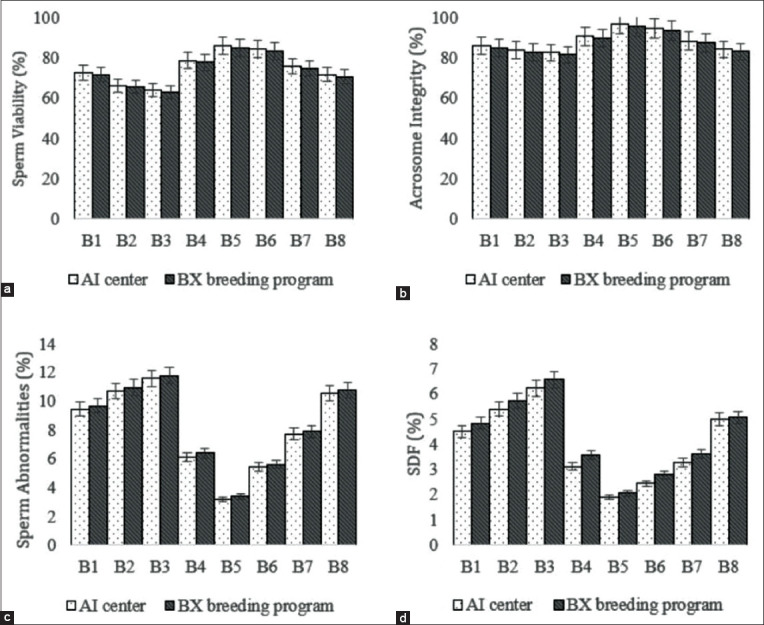
The results of the assessment of sperm viability (a), acrosome integrity (b), sperm abnormality (c), and sperm DNA fragmentation (d) in frozen semen before and after distribution in the Brahman Cross breeding program.

Sperm viability also showed no statistically significant difference between frozen-thawed semen before and after distribution (p>0.05). (Figure-1) Sperm viability was in the range of 62-85% (Figure-2). Results of the analysis on acrosome integrity and sperm abnormality (Figure-1) also did not show any significant differences (p>0.05) in frozen-thawed semen between the AI center and BX breeding programs, with ranges of 81-95% and 4-9%, respectively (Figure-2). Less than 6% of sperm in frozen-thawed semen after distribution in the BX breeding program underwent DNA fragmentation (Figure-1), and there was no significant difference (p>0.05) with frozen semen in the AI center (Figure-2).

**Figure-3:**
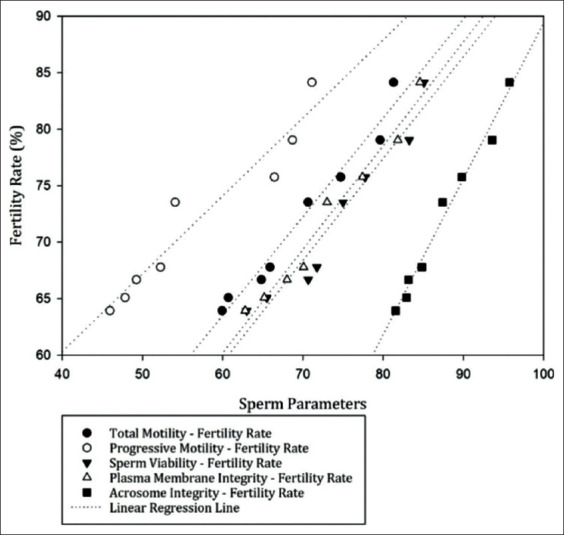
The relationship between total and progressive motility, sperm viability, plasma membrane integrity, and acrosome integrity with fertility rate (p<0.05) in frozen-thawed semen after distribution in the BX breeding program.

**Figure-4:**
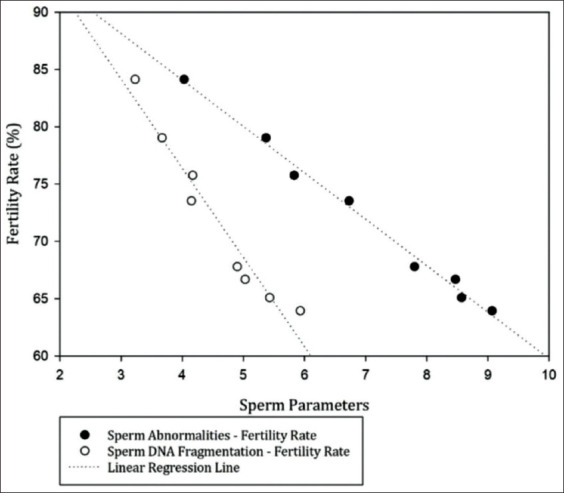
The relationship between sperm abnormalities and sperm DNA fragmentation with fertility rate (p<0.05) in frozen semen after distribution in the BX breeding program.

The outcome of the fertility analysis using the CR of the 502 insemination services in 478 cows in the BX breeding program for each bull (B1-B8), respectively, was 67.78%, 65.08%, 63.93%, 75.76%, 84.13%, 79.03%, 73.53%, and 66.67% (Table-2). TM (r=0.986), PM (r=0.961), sperm viability (r=0.971), PMI (r=0.986), and acrosome integrity (r=0.992) all had a significant positive correlation with fertility rate (p<0.05) (Figure-3); while sperm abnormalities (r=−0.996) and sperm DNA fragmentation (r=−0.975) are negatively correlated (p<0.05) with fertility rate (Figure-4).

**Table-2:** The fertility rate derived from the conception rate (%) of the insemination of 478 cows and 502 artificial insemination services in the Brahman Cross breeding program for each bull.

Bulls (n=8)	Number of services (straw)	Number of cows	Conception rate (%)
B1	102	90	67.78
B2	66	63	65.08
B3	61	61	63.93
B4	68	66	75.76
B5	64	63	84.13
B6	67	62	79.03
B7	34	34	73.53
B8	40	39	66.67

B1-B8=Bull-1…bull-8

## Discussion

Overall, there was no significant difference in the quality of frozen-thawed semen at the production site (AI center) and after being distributed to the BX breeding program (p>0.05). Changes that may occur in the quality of frozen-thawed semen, such as impairment in some parameters (total and PM, viability, PMI, and acrosome integrity), increased sperm abnormalities and fragmentation of sperm DNA are likely caused by temperature changes that occur during the process of transferring frozen-thawed semen samples from the main storage container to smaller containers for shipment. Carrell *et al*. [19] suggested that the transfer of frozen-thawed semen samples between containers during distribution or delivery between locations will cause changes in temperature over a short period. This is often ignored, but it can cause damage and decrease sperm quality. Carrell *et al*. [19] also argued that storage containers, higher frequency of transportation activities, and trip duration during sample distribution would cause temperature changes and therefore have an impact on the quality of the sample. On the contrary, Til *et al*. [20] reported that the distribution or transportation of frozen semen would not affect the quality of the semen as long as it remained immersed in liquid nitrogen at a temperature of −196°C. This suggests that the distribution of frozen semen in the current study adhered to these measures and as a result, we observed no alterations in the quality of the frozen-thawed semen. We have provided further evidence that as long as temperature during transit is maintained at −196°C and the integrity of the containers is maintained then no differences in semen quality will be observed between the production point and the field. However, other than handling and container management factors during distribution, several other factors that can affect the quality of semen must also be considered and regulated. These include genetics [14,15], breed, age [16], environment [14,15], and various management factors, including nutrition and semen collection management until completion of the freezing process to when the samples are ready for distribution [2,24].

Fertility rates are more important in bulls than in cows, because one bull can be used to breed up to 40 cows by natural mating, or potentially hundreds of thousands through AI programs [27]. Dalton [28] revealed that the maximum fertility rate can be obtained from cattle with high fertility coupled with good, high-quality semen. The fertility rate based on the CR in the BX breeding program is 63-79%. Diskin [29] revealed that a good CR is at least 60-70%, while Butler [14] suggests CRs of up to70% of all first-inseminated cows/heifers. The high CRs achieved in the current study are likely to be a direct result of good management practices implemented in the BX breeding program. The handling of frozen semen from distribution to storage is well controlled to maintain semen quality, sound breeding practices, and constant expert supervision and monitoring of cow reproductive health are all strategies employed in the BX breeding program. This particular example shows the level of management seen on several other farms in Indonesia that help maintain high pregnancy outcomes.

Sperm motility inherently determines sperm’s ability to fertilize oocytes and is thus an important characteristic in the assessment of semen quality before and after thawing [30]. Cojkic *et al*. [31] suggested that sperm motility is very closely related to the success of fertilization, and the findings of the current study support this view (Figure-3). The total and PM of sperm after being distributed in the BX breeding program show values of 45.93±0.55%-71.12±2.53% (Table-1), which are above the recommended minimum of 40% of sperm showing good and PM after freezing and thawing, suggested by Zewdie *et al*. [32].

Furthermore, positively correlated with fertility is sperm viability, which is one of the requirements for oocyte fertilization (Figure-3) [10,31]. The viability of frozen-thawed semen after distribution in the BX breeding program was between 62.98±0.01% and 85.08±0.04% (Figure-2). According to Rasul *et al*. [33], as much as, 50% of sperm viability is lost after the freezing process. Schüffner *et al*. [34] also added that the level of motility and viability of sperm cells after the cryopreservation process can decrease by 25-75%. This occurs because of the pressure that occurs in sperm cells during the cryopreservation process; this is caused by cellular dehydration, high solute concentrations, and changes in PMI that result in functional changes and structural damage [31].

PMI is essential for optimizing sperm function [35] because only sperm with intact plasma membranes can survive the series of complex changes in the female reproductive tract and be able to fertilize the oocyte. The integrity of the plasma membrane and acrosome in the study had significant positive correlations with fertility rates (Figure-3). This provides evidence that sperm with intact plasma membranes and intact acrosomes have higher fertilization abilities and high CRs. As highlighted by Graham and Moce [36], an intact plasma membrane and an undamaged acrosome are vital in the capacitation process, the acrosomal reaction, and oocyte fertilization. The PMI in frozen-thawed semen after being distributed in the BX breeding program was 62.79±0.01%-84.58±0.01% (Table-1), while acrosome integrity was 81.58±0.01%-95.75±0.01% (Figure-2). Given that the minimum standard quality of post-thawing frozen-thawed semen for sperm PMI is ≥40% and acrosome integrity is ≥65% [37], the integrity of the plasma membrane and acrosome membranes in the current study reflects semen that is considered optimal in its expected fertilizing ability.

High sperm abnormalities will have a negative impact and affect fertility [38] (Figure-4). Total sperm abnormalities in frozen-thawed semen after distribution in the BX breeding program were 4.03±0.01%-9.07±0.01% (Figure-2). Ball and Peters [39] reported that bulls with total sperm abnormalities >17% would have low fertilization rates. Patel *et al*. [38] also reported that the maximum abnormalities of sperm in cattle were <20%, of which bulls with good fertility had a total abnormality ranging from 8% to 12%. The sperm abnormalities in the current study are relatively low, pointing to frozen-thawed semen of good quality.

DNA fragmentation has an association with fertility (Figure-4); high DNA damage will have a negative impact on embryonic development and decrease fertility [7]. The results of the DNA fragmentation analysis of frozen-thawed semen after field distribution were 3.23±0.001%-5.93±0.01% (Figure-2). The fragmentation observed in bull sperm DNA is still within acceptable limits and can still produce high fertility. Bochenek *et al*. [40] found that fertility decreases in males whose chromatin is damaged or whose DNA fragmentation reached 10%. Other studies also mention a 15% or less DNA fragmentation in the semen as still being normal, while 15-25% will reduce fertility and semen production. DNA damage above 25% is considered infertile [41].

## Conclusion

The study shows that to achieve the maximum and optimal fertility in bulls in an AI program, the quality of frozen-thawed semen should be considered in its entirety. This can certainly be achieved if the handling of frozen semen throughout the distribution channel is carried out effectively. However, various other factors that can also affect semen quality such as genetics, breed, age, environment, and various management factors including nutrition, semen collection until the freezing process is complete and ready to be distributed should be considered. In addition, breeding management factors must also be factored in and controlled properly. Additional variables such as frozen semen storage at various temperatures, transportation type, storage times after distribution, and distribution locations with various breeding management conditions will enrich research related to the quality of bull semen after distribution on the field in the future.

## Authors’ Contributions

BPP and MA conceptualized and designed this study. BPP performed the experiment under the guidance of MA, IS, and YY. BPP analyzed the results, literature search, and wrote the first manuscript draft. MA, IS, and YY edited and revised the manuscript. All authors critically read, reviewed, and approved the final manuscript.

## References

[1] Bearden H.J, Fuquay J.W (2004). Applied Animal Reproduction.

[2] Kebede A (2018). Review on factors affecting success of artificial insemination. Int. J. Curr. Res. Aca. Rev.

[3] Haile-Mariam M, Pryce J (2019). Advances in Breeding of Dairy Cattle:Advances in Dairy Cattle Breeding to Improve Fertility/Reproductive Efficiency.

[4] Pardede B.P, Tamba B, Sutrisnak S, Wisana I.K.K, Rahardjo H.B, Agil M, Yusuf T.L (2018). Production Trait of Crossbreed Cattle and Reproductive Disorders in Brahman Cross (BX) Breeding Program at PT Lembu Jantan Perkasa.

[5] Dogan S, Vargovic P, Oliveira R, Belser L.E, Kaya A, Moura A, Sutovsky P, Parrish J, Topper E, Memili E (2015). Sperm protamine-status correlates to the fertility of breeding bulls. Biol. Reprod.

[6] Mekonnen T, Bekana M, Abayneh T (2010). Reproductive performance and efficiency of artificial insemination smallholder dairy cows/heifers in and around Arsi-Negelle, Ethiopia. Livestock Res. Rural Dev.

[7] Kumaresan A, Johannisson A, Al-Essawe E.M, Morrell J.M (2017). Sperm viability, reactive oxygen species, and DNA fragmentation index combined can discriminate between above-and below-average fertility bulls. J. Dairy Sci.

[8] Sitali M.C, Mwanza A.M, Mwaanga E.S, Parsons I.R, Parsons N.J (2017). Sperm morphology and sperm quality of bulls raised on commercial farms in Zambia. Int. J. Adv. Bio. Res.

[9] Sellem E, Broekhuijse M, Chevrier L, Camugli S, Schmitt E, Schibler L, Koenen E (2015). Use of combinations of *in vitro* quality assessments to predict fertility of bovine semen. Theriogenology.

[10] Morrell J.M, Rodriguez-Martinez H (2009). Biomimetic techniques for improving sperm quality in animal breeding:A review. Open Androl. J.

[11] Barrier-Battut I, Kempfer A, Becker J, Lebailly L, Camugli S, Chevrier L (2016). Development of a new fertility prediction model for stallion semen, including flow cytometry. Theriogenology.

[12] Patil S, Kumar P, Singh G, Bala R, Jerome A, Patil C.S, Kumar D, Singh S, Sharma R.K (2020). Semen dilution effect on sperm variables and conception rate in buffalo. Anim. Reprod. Sci.

[13] Rusdiana S, Soeharsono S (2018). SIWAB program to improve cattle population and economics value for the business economics. Forum Penelitian Agro Ekonomi.

[14] Butler S (2014). Dairy Cow Reproduction.

[15] Pardede B.P, Supriatna I, Agil M (2020). Protamine and other proteins in sperm and seminal plasma as molecular markers of bull fertility. Vet. World.

[16] Pardede B.P, Supriatna I, Yudi Y, Agil M (2020). Decreased bull fertility:Age-related changes in sperm motility and DNA fragmentation. E3S Web Conf.

[17] McDonald C.A, Valluzo L, Chuang, L, Poleshchuk F, Copperman A.B, Barritt J (2011). Nitrogen vapor shipment of vitrified oocytes:Time for caution. Fertil. Steril.

[18] Parmegiani L, Maccarini A.M, Rastellini A, Bernardi S, Troilo E, Arnone A, Lanzilotti S, Filicori M (2017). Oocyte vitrification/storage/handling/transportation/warming, effect on survival and clinical results in donation programmes. Curr. Trends Clin. Embryol.

[19] Carrell D.T, Wilcox A.L, Urry R.L (1996). Effect of fluctuations in temperature encountered during handling and shipment of human cryopreserved semen. Androgolia.

[20] Til D, Amaral V.L, Salvador R.A, Senn A, de Paula T.S (2016). The effects of storing and transporting cryopreserved semen. JBRA Assisted. Reprod.

[21] Ramírez-Reveco A, Hernandez J.L, Aros P (2016). Long-term Storing of Frozen Semen at -196°C Does not Affect the Post-thaw Sperm Quality of Bull Semen. OpenIntech, London UK.

[22] Jeyendran R.S, Vander-Van H.H, Perez-Pelaez M, Crabo B.G, Zaneveld L.J (1984). Development of an assay to assess the functional integrity of the human sperm membrane and its relationship to other semen characteristics. J. Reprod. Fertil.

[23] Dias T.R, Cho C.L, Agarwal A, Sperm assessment:Traditional approaches and their indicative value (2019). *In vitro* Fertilization:A Textbook of Current and Emerging Methods and Devices.

[24] Kanwa L, Hussain S.A, Ahmed H, Hussain A, Andrabi S.M.H (2020). Alpha-tocopheryl succinate in extender improves the post thaw quality of water buffalo spermatozoa. Cryo. Lett.

[25] Morrell J, Valeanu A.S, Lundeheim N, Johannisson A (2018). Sperm quality in frozen beef and dairy bull semen. Acta Vet. Scand.

[26] Baskaran S, Cho C.L, Agarwal A (2019). Role of Sperm DNA Damage in Male Infertility Assessment:Male Infertility in Reproductive Medicine:Diagnosis and Management. CRC Press USA.

[27] Kastelic J.P (2013). Male involvement in fertility and factors affecting semen quality in bulls. Anim. Front.

[28] Dalton J.C (2011). Semen Quality Factors Associated with Fertility.

[29] Diskin M.G (2014). Achieving High Reproductive Performance in Beef Herds.

[30] Puglisi R, Pozzi A, Foglio L, Spano M, Eleuteri P, Grollino M.G, Bongioni G, Galli A (2012). The usefulness of combining traditional sperm assessments with *in vitro* heterospermic insemination to identify bulls of low fertility as estimated *in vivo*. Anim. Reprod. Sci.

[31] Cojkic A, Dimitrijevic V, Savic M, Jeremic I, Vukovic D, Cobanvic N, Obradovic S, Petrujkic B.T (2017). The correlation between selected computer-assisted sperm analysis parameters and bull fertility. Vet. Arhiv.

[32] Zewdie E, Deneke N, Fikre-Mariam D, Chaka E, Haile-Mariam D, Mussa A (2005). Guidelines and Procedures on Bovine Semen Production.

[33] Rasul Z, Ahmed N, Anzar M (2007). Antagonist effect of DMSO on the cryoprotection ability of glycerol during cryopreservation of buffalo sperm. Theriogenology.

[34] Schüffner A, Morshedi M, Oehninger S, Carvalho N.S, Oliveira M.C, Placido T, Urbanetz A.A (2008). Apoptosis and lipid peroxidation before and after cryopreservation. Reprod. Clim.

[35] Matabane M.B, Thomas R, Netshirovha T.R, Tsatsimpe M, Ng'ambi J, Nephawe K.A, Nedambales T.L (2017). Relationship between sperm plasma membrane integrity and morphology and fertility following artificial insemination. South Afr. J. Anim. Sci.

[36] Graham J.K, Mocé E (2005). Fertility evaluation of frozen-thawed semen. Theriogenology.

[37] India Agriculture Ministry (2014). Compendium of Minimum Standards of Protocol and Standard Operating Procedures for Bovine Breeding.

[38] Patel G.K, Haque N, Madhavatar M, Chaudhari A.K, Patel D.K, Bhalakiya N, Jamnesha N, Patel P, Kumar R (2017). Artificial insemination:A tool to improve livestock productivity. J. Pharm. Phytochem.

[39] Ball P.J, Peters A.R (2004). Reproduction in Cattle.

[40] Bochenek M, Smorag Z, Pilch J (2001). Sperm chromatin structure assay of bulls qualified for artificial insemination. Theriogenology.

[41] Larson-Cook K.L, Brannian J.D, Hansen K.A, Kasperson K.M, Aamold E.T, Evenson D.P (2003). Relationship between the outcomes of assisted reproductive techniques and sperm DNA fragmentation as measured by the sperm chromatin structure assay. Fertil. Steril.

